# Supplementary material for: Nucleosome-directed replication origin licensing independent of a consensus DNA sequence
Source: Nat Commun. 2022 Aug 23;13:4947. doi: 10.1038/s41467-022-32657-7 (PMC9399094; doi:10.1038/s41467-022-32657-7)
Supplement: Supplementary file 1 — Supplementary Information [file 41467_2022_32657_MOESM1_ESM.pdf]

SUPPLEMENTARY TABLE 1

Sequence Description	Source
ARS1-containing DNA sequence inserted into λ DNA (ARS1 sequence is underlined; B3, B2, B1 and ACS elements in bold):<br><br>5’-TCGAGAAGCAGGTGGGACAGGTGAACTTTTGGATTGGAACTCGATTTCTGACTG<br>GGTTGGAAGGCAAGAGAGCCCCGAAAGCTTACATTTTATGTTAGCTGGTGGACTG<br>ACGCCAGAAAATGTTGGTGATGCGCTTAGATTAAATGGCGTTATTGGTGTTGATG<br>TAAGCGGAGGTGTGGAGACAAATGGTGTAAAAGACTCTAACAAAATAGCA**AATT**<br>**TCGTCAAAAATGCT**AAGAAATAGGTTATTACTGAGTAGTATTT**ATTTAAGTATT**G<br>TTTGTGCACTTGCCTGCAGGCCTTT**TGAAAAGCAAGCA**TAAAAG**ATCTAAACATA**<br>**AAATCT**GTAAAATAACAAGATGTAAAGATAATGCTAAATCATTTGGCTTTTTGATT<br>GATTGTACAGGAAAATATACATCGCAGGGGGTTGACTTTTACCATTTCACCGCAA<br>TGGAATCAAACTTGTTGAAGAGAATGTTCACAGGCGCATACGCTACAATGACCCG<br>ATTCTTG-3’	This paper

SUPPLEMENTARY FIGURES

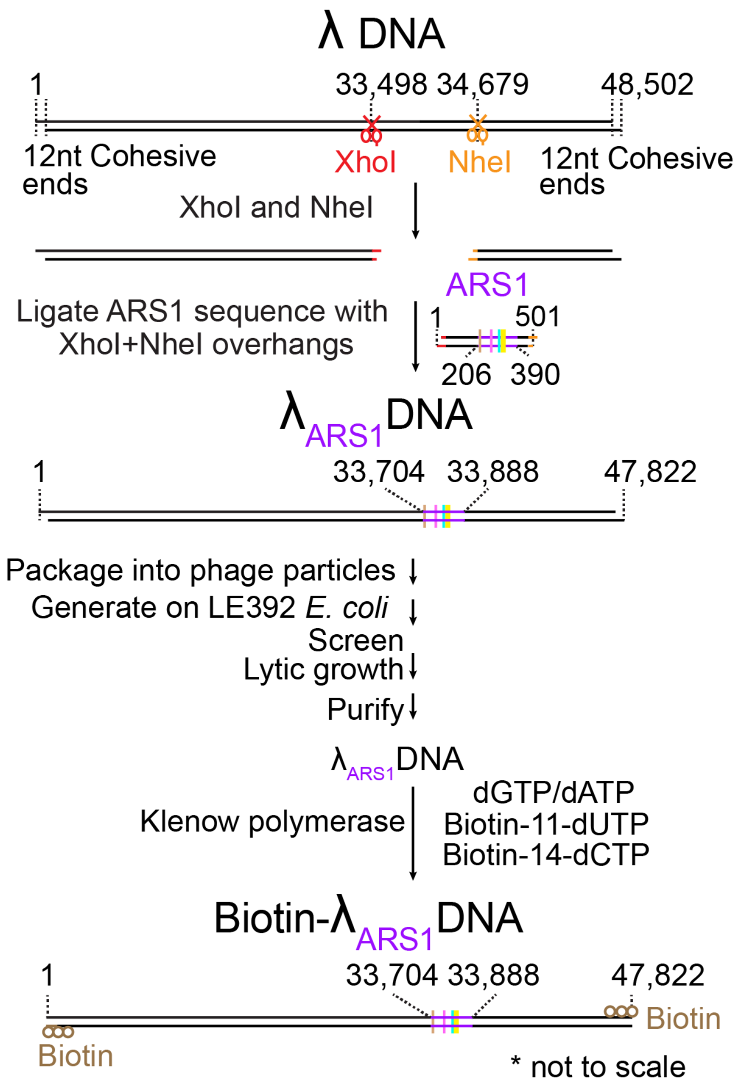

Supplementary Figure 1. Workflow for generating the biotinylated λ_{ARS1} DNA

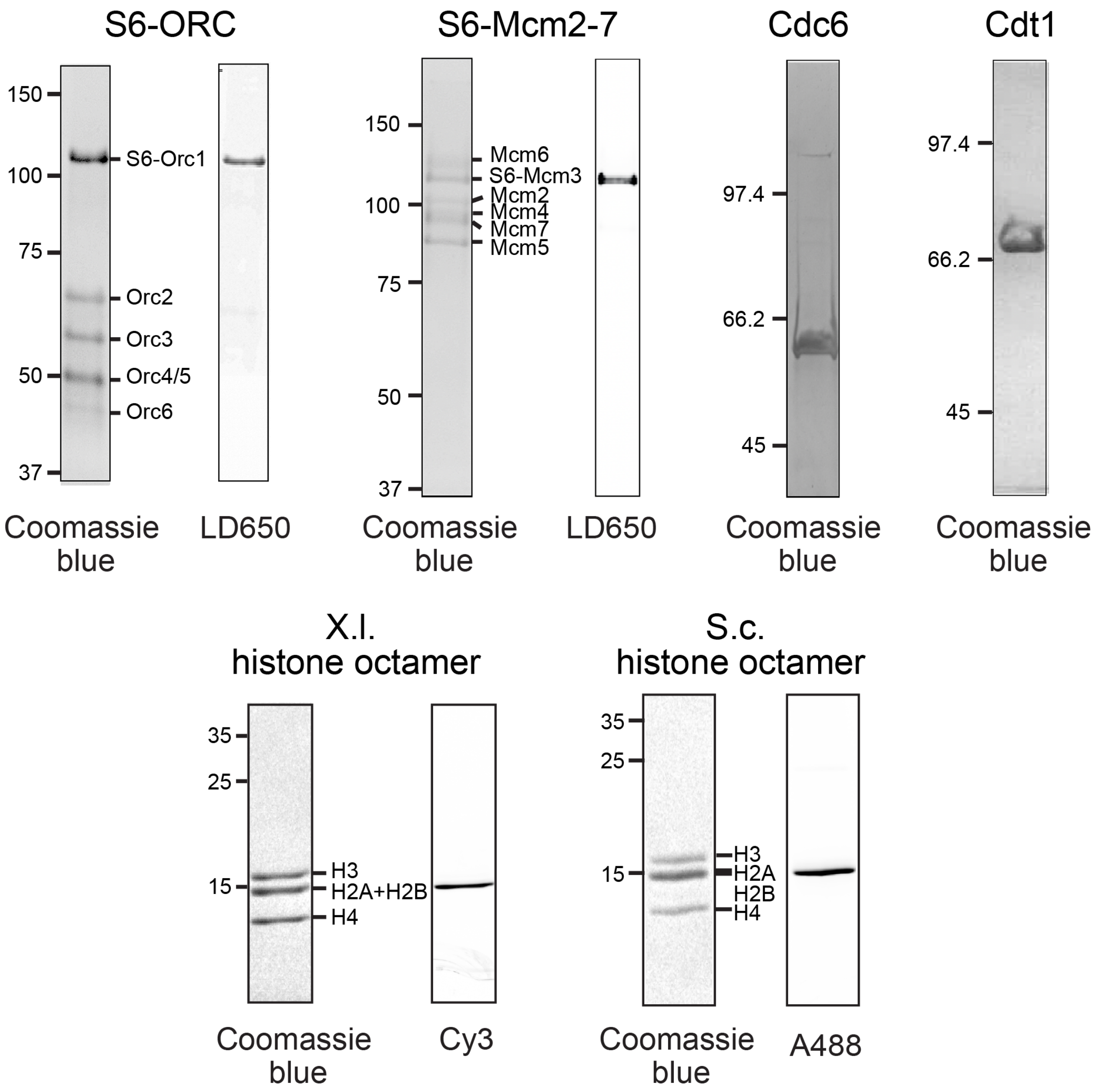

Supplementary Figure 2. SDS-PAGE gels for proteins used in this work and fluorescence images for ORC, Mcm2-7 and histone octamers. Shown are representative results from at least three independent sample preparations. Source data are provided as a Source Data file.

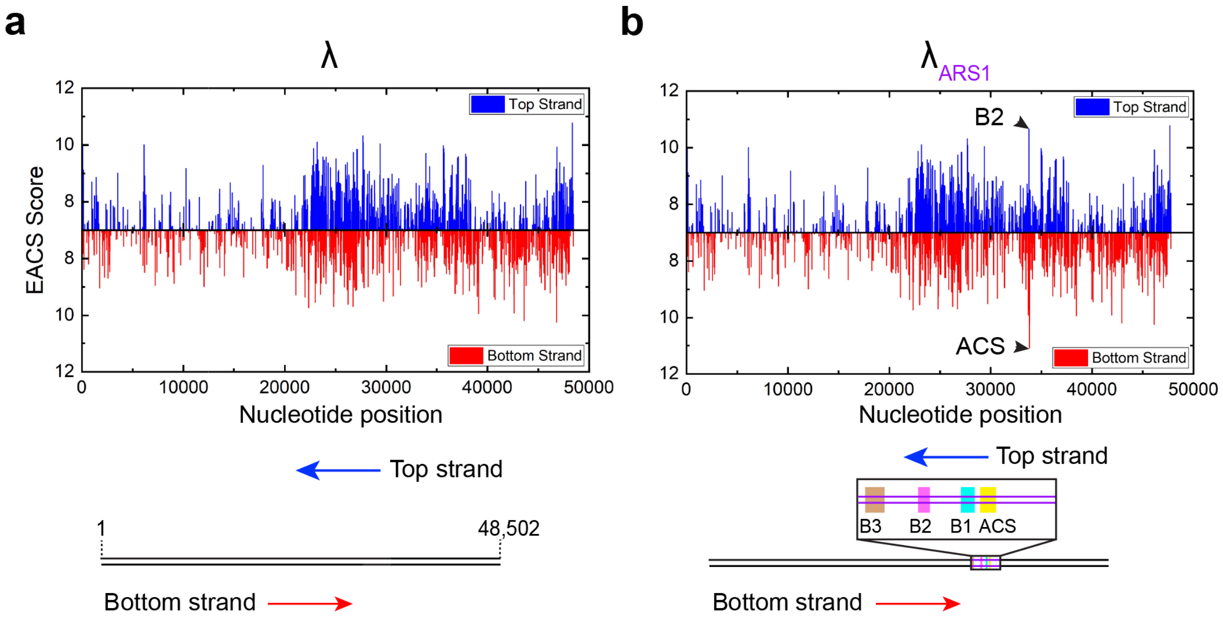

Supplementary Figure 3. Analysis of potential ACS motifs within λ DNA

a Extended ACS (EACS) scores for the native λ genomic DNA using a 17-bp position weight matrix (PWM) generated from multiple sequence alignment of functional ARS elements identified in *S. cerevisiae* and previously used to score potential ORC binding sites in a given DNA sequence [1-2] . **b** EACS scores for the engineered λ_{ARS1} DNA. The highest scores correspond to the ARS1 elements (black arrowheads) inserted in the λ template.

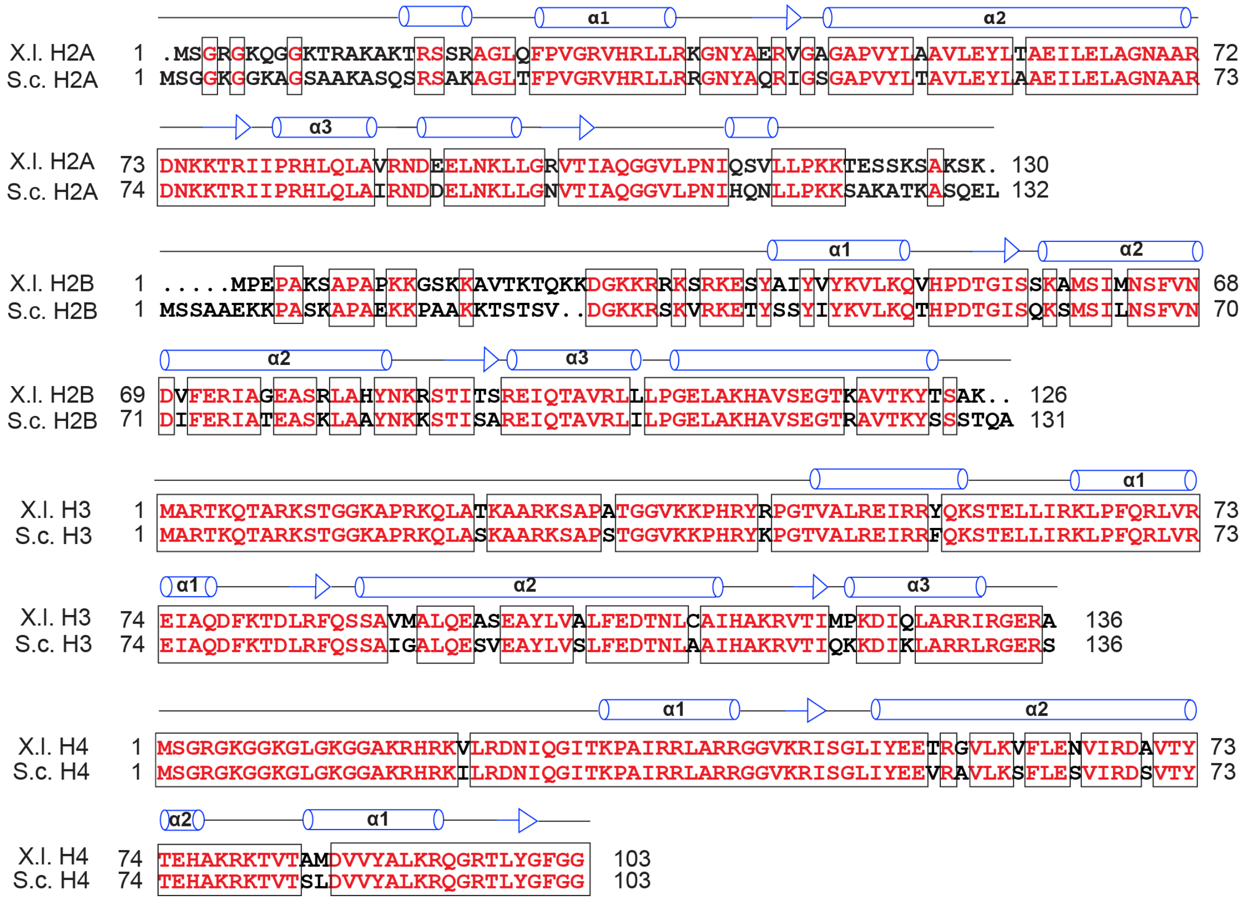

Supplementary Figure 4. Sequence alignment of *Xenopus laevis* and *Saccharomyces cerevisiae* histones. Identical residues are colored in red.

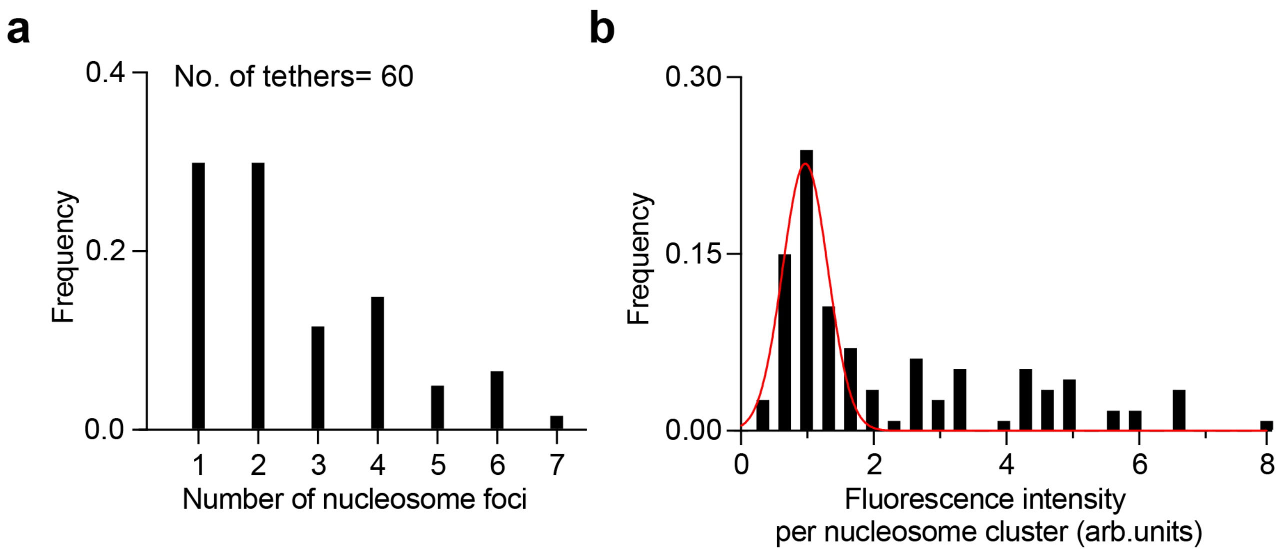

Supplementary Figure 5. Evaluation of nucleosome loading on tethered DNA

a Distribution of the number of fluorescently labeled nucleosome foci per DNA tether. **b** Distribution of the fluorescence intensity per nucleosome cluster. The main peak (red curve) represents the mononucleosome population. Source data are provided as a Source Data file.

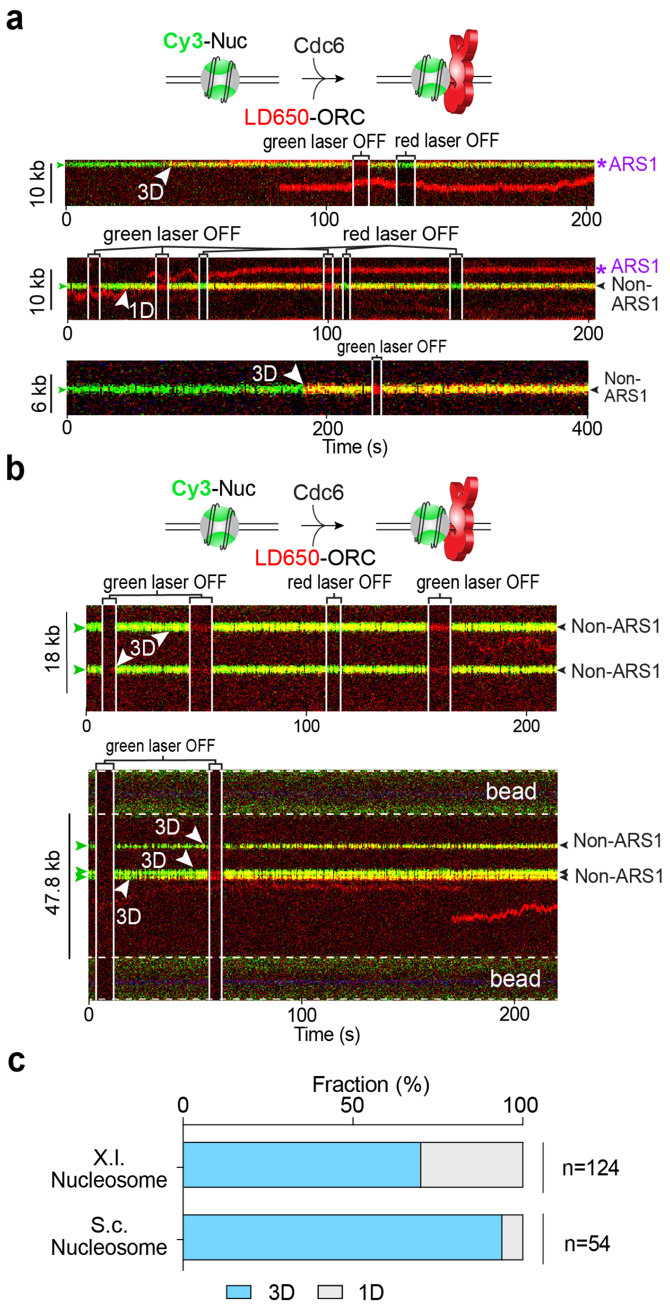

Supplementary Figure 6. Analysis of the search mode used by ORC to target nucleosomes

a Three example kymographs showing LD650-labeled ORC (red) stably associated with Cy3-labeled X.l. nucleosomes (green). White arrowheads indicate the time when ORC arrived at the nucleosomal site (ARS1 or non-ARS1) via three-dimensional (3D) or one-dimensional (1D) search. Green arrowheads indicate the positions of nucleosomes. **b** Two example kymographs showing LD650-labeled ORC (red) binding to Cy3-labeled S.c. nucleosomes (green). **c** Fraction of ORC molecules that were observed to target a nucleosome (X.l. or S.c.) by 3D or 1D search. n indicates the number of events analyzed for each condition. Source data are provided as a Source Data file.

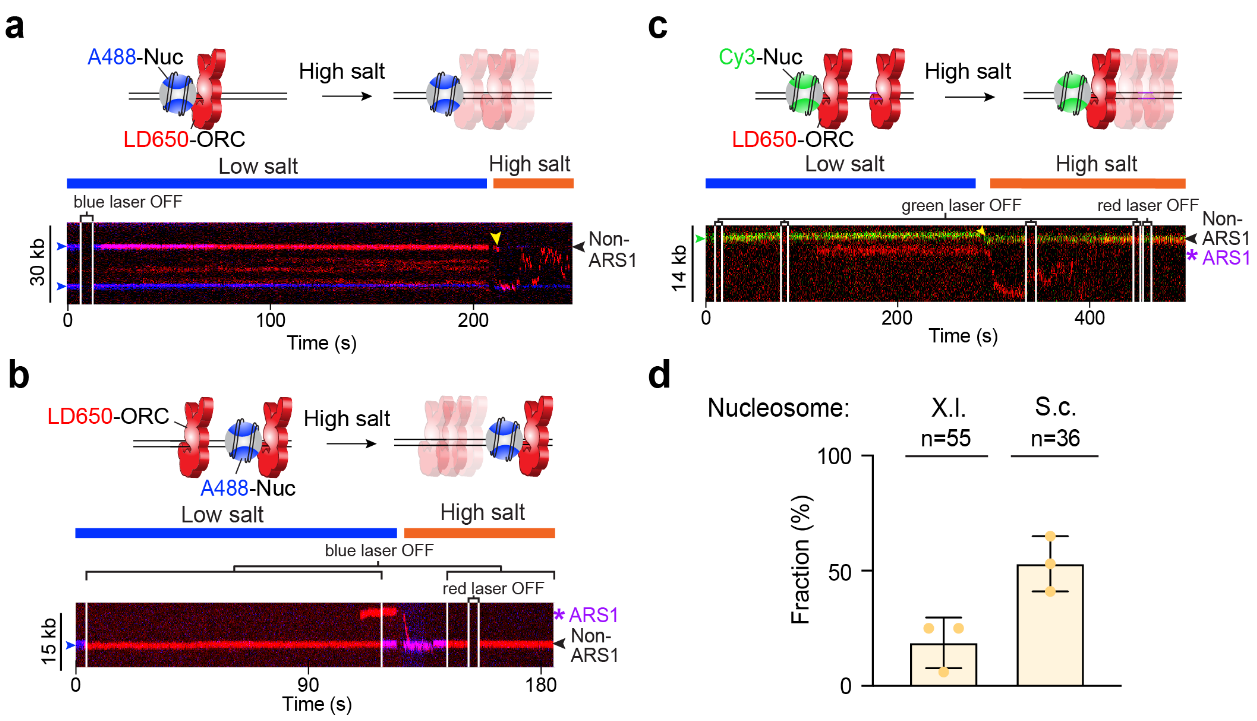

Supplementary Figure 7. The fraction of ORC that remained associated with nucleosomes at high salt
a Cartoon and an example kymograph showing ORC (red) colocalized with a S.c. nucleosome (blue) in a low-salt buffer but departed from the nucleosome and underwent diffusive motions on DNA upon high-salt wash (yellow arrowhead). **b** Cartoon and an example kymograph showing an ORC molecule (red) colocalized with a S.c. nucleosome (blue) and remained bound to the nucleosome at high salt. Another ORC bound at the ARS1 DNA position underwent diffusion upon high-salt wash. **c** Cartoon and an example kymograph showing an ORC molecule (red) colocalized with a Cy3-labeled X.l. nucleosome (green) and remained bound to it at high salt. Another ORC bound at the ARS1 DNA position underwent diffusion upon high-salt wash (yellow arrowhead). **d** Fraction of ORC molecules that were observed to depart from a nucleosomal site (X.l. vs. S.c.) upon high-salt wash. n indicates the number of ORC molecules analyzed for each condition. Data are presented as mean values +/- SD from three independent experiments. In all experiments, ORC was first incubated with tethered nucleosomal DNA at low salt in the presence of Cdc6; then the whole assembly was moved to another channel containing high salt. Source data are provided as a Source Data file.

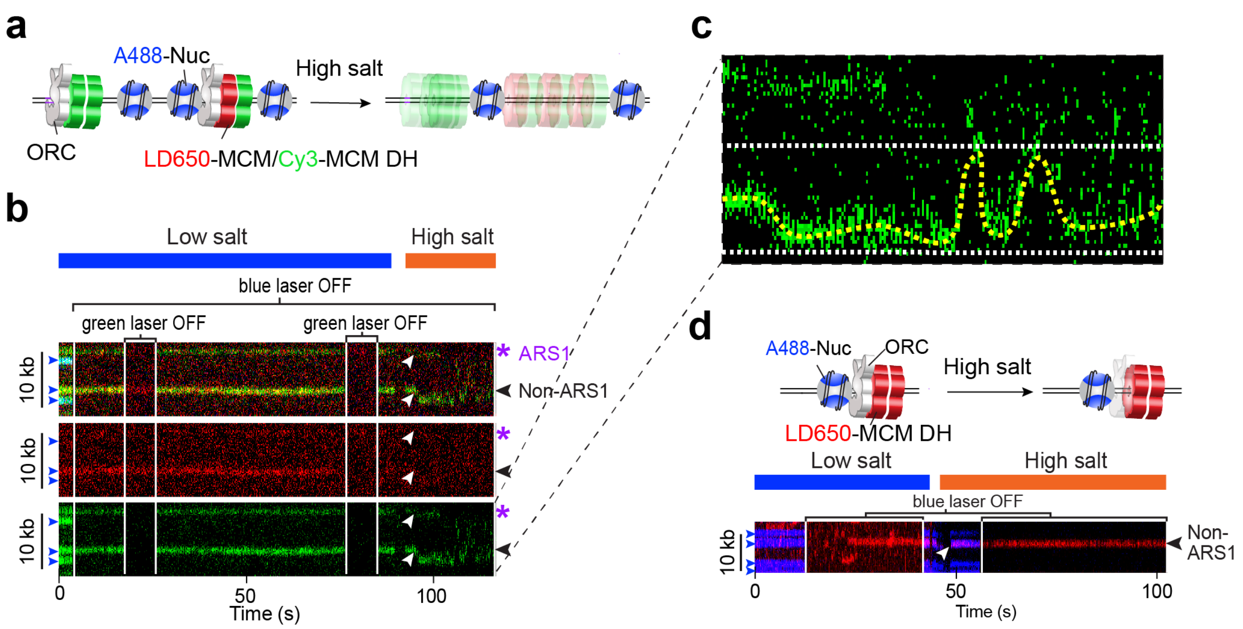

Supplementary Figure 8. Additional examples showing the behavior of nucleosome-bound MCM upon high-salt wash

a Cartoon illustrating that MCM DHs undergo diffusive motions on DNA upon high-salt wash. The reaction also contained unlabeled ORC, Cdc6 and Cdt1. **b** Kymograph of two MCM complexes on a DNA tether loaded with nucleosomes. Individual red and green channels are shown underneath the merged image. The MCM at the non-ARS1 nucleosome site was composed of a green MCM and a red MCM (photobleached before the second "green laser off" window). Blue arrowheads indicate the positions of nucleosomes, which were only visualized at the very initial stage of data collection before the blue laser was turned off (for clarity). Upon entering the high-salt channel, both MCM complexes started sliding on DNA (white arrowheads), as expected for a loaded MCM DH that encircles DNA. **c** Zoomed-in view of MCM DH sliding in high salt. The yellow dotted line traces the trajectory of MCM sliding that is confined between two nucleosomes (white dotted lines). **d** Cartoon and kymograph showing that an MCM (red) colocalized with a non-ARS1 nucleosome (blue) in a low-salt buffer and remained bound to the nucleosome at high salt.

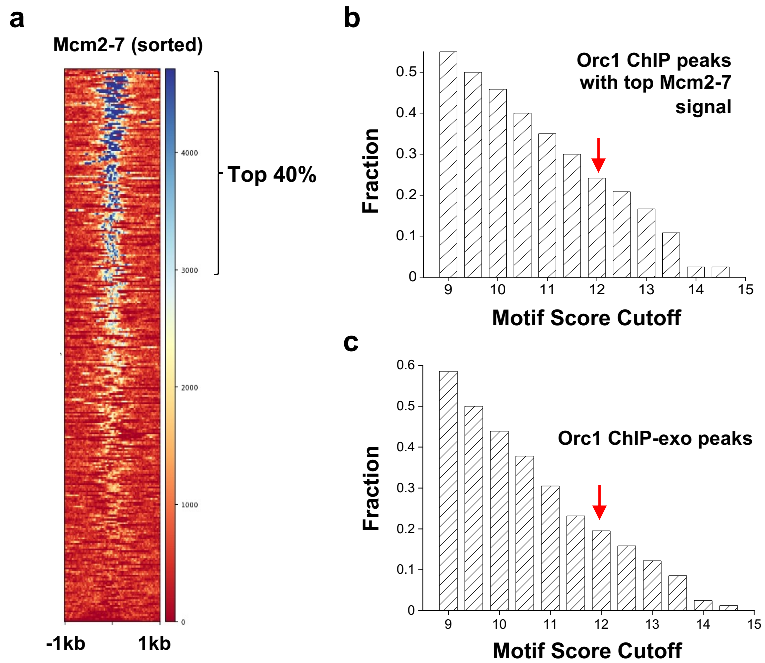

Supplementary Figure 9. Additional analysis of genome-wide ORC/MCM localization

a Heatmap of sorted Mcm2-7 ChIP signals among the 295 Orc1 ChIP peaks. The top 40% of these sites are included in the analysis in **b**. **b** Fraction of sequences that contain motifs above a certain threshold (varying from 9 to 15). These sequences are extracted from the Orc1 peaks with high Mcm2-7 ChIP signals. The red arrow represents the recommended cutoff of 11.9. **c** Same as in **b** except that the sequences are extracted from Orc1 ChIP-exo peaks (n = 81).

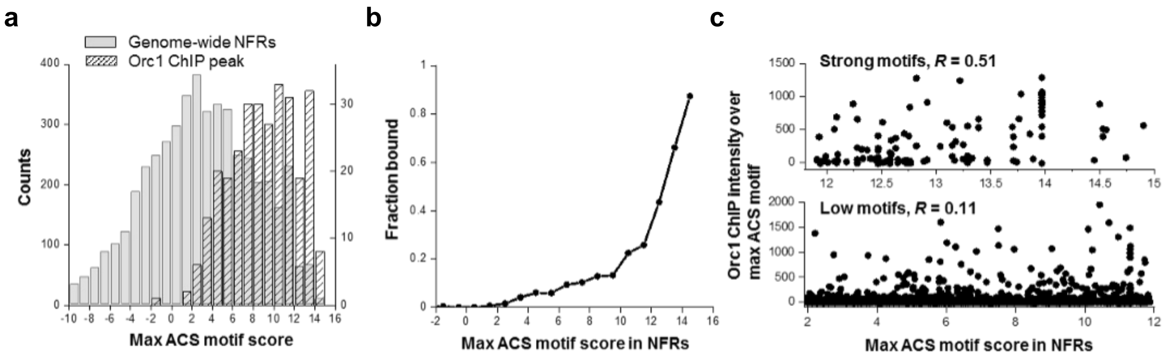

Supplementary Figure 10. Additional genome-wide analysis of ORC binding and ACS motif scores in NFRs

a ACS motif scores in genome-wide NFRs (grey bars) versus those in underneath Orc1 ChIP peaks (black bars). ORC binding frequently occurs over motifs with score < 11, thus motif alone cannot predict the selectivity of ORC binding in NFR. **b** Percentage of ACS motifs in NFRs that associate with Orc1 ChIP peaks as a function of the motif score. Motif plays a strong role in directing ORC binding in NFR only when it is a near-perfect match to the consensus. **c** Correlation of Orc1 ChIP peak intensity in NFRs with ACS motif score in the high score range (PWM >= 11.9) or low score range (PWM < 11.9). For strong motifs, ORC binding displays a moderate correlation with the motif score (Pearson correlation coefficient R = 0.51), while for the weak ones, the correlation is very weak (R = 0.11).

REFERENCES

1. Coster, G. & Diffley, J.F.X. Bidirectional eukaryotic DNA replication is established by quasi-symmetrical helicase loading. *Science* **357**, 314-318 (2017).
2. Liachko, I., Youngblood, R.A., Keich, U. & Dunham, M.J. High-resolution mapping, characterization, and optimization of autonomously replicating sequences in yeast. *Genome Res* **23**, 698-704 (2013).
