# Supplementary material for: Nucleosome-directed replication origin licensing independent of a consensus DNA sequence
Source: Nat Commun. 2022 Aug 23;13:4947. doi: 10.1038/s41467-022-32657-7 (PMC9399094; doi:10.1038/s41467-022-32657-7)
Supplement: Supplementary file 3 — Description of Additional Supplementary File [file 41467_2022_32657_MOESM3_ESM.pdf]

Description of Additional Source Data Files

Supplementary Data File 1. - Legend: “The correspondence between identified Orc1 peaks and previously annotated ARSs.”

Source Data Legends

Source Data Fig. 2. - Statistical source data for Fig. 2d and 2e.

Source Data Fig. 3. - Statistical source data for Fig. 3c, 3e, 3f and 3i.

Source Data Fig. 4.- Statistical source data for Fig. 4c and 4d.

Source Data Fig. 5.- Statistical source data for Fig. 5c, 5d and 5e.

Source Data Fig. 6.- Statistical source data for Fig. 6c.

Source Data Supp Fig. 2.- Full scan images for Supp. Fig. 2.

Source Data Supp Fig. 5. - Statistical source data for Supp. Fig. 5.

Source Data Supp Fig. 6. - Statistical source data for Supp. Fig. 6c.

Source Data Supp Fig. 7. - Statistical source data for Supp. Fig. 7d.
